# Management of tic disorders in a community child and adolescent services

**DOI:** 10.1192/j.eurpsy.2026.10559

**Published:** 2026-07-31

**Authors:** S. O. Oranu, T. Hossain, A. Segal, B. C. Poornamodan

**Affiliations:** Child and adolescent psychiatry, North Staffordshire Combined NHS Trust, Stoke on Trent, United Kingdom

## Abstract

**Introduction:**

Tic disorders are neurodevelopmental conditions frequently presenting in childhood and adolescence. Management often requires a multidisciplinary approach, incorporating neurology, psychiatry and psychology. A structured care pathway for the management of Tic disorders in the community was introduced in North Staffordshire Child and Adolescent Mental health services in 2023. This was aimed to standardise assessment, provide psychoeducation, offer group therapy interventions and medication. Understanding real-world pathway performance is essential to identify service strengths and gaps.

**Objectives:**

A)Map referral patterns to the TIC Pathway and pre-referral neurology assessments. B) Evaluate waiting times from referral to initial assessment. C) Assess the provision of psychoeducation and therapy. D)Examine clinical outcomes following therapeutic interventions. E)Review psychiatric input within the pathway.

**Methods:**

Patients with Tic disorder were identified from case notes. Sample included young people who were accepted into the service between April 2024 and March 2025.Data was collected using a semi-structured questionnaire.

**Results:**

26 patients (n=26) were identified as having Tic disorder across all the community CAMHS teams.

**Referral sources:** Most patients were referred by their GPs (62%) and paediatricians (19%). School nurses and self referrals constituted the rest.

**Neurology assessment prior to referral:** In 50% of the cases, this was not clearly documented. Amongst those recorded, this was completed in 77% of cases.

**Time from referral to initial assessment: 54% of** young people had to wait for more than 16 weeks for an initial assessment.

**Provision of Psychoeducation after initial assessment:** Only 2 patients received psychoeducation. In 88% (23 cases) this information was not recorded.

**Psychiatric co-morbidities identified at initial assessment:** 65% of patients had a psychiatric comorbidity. ASD was the commonest comorbidity (n = 17), with ADHD(6), anxiety (4) and OCD (2) making up the others.

**Therapy:** Nearly all patients were offered therapy (96%) following initial assessment. The most common intervention was the TREAT group (65%) and 31% had both TREAT group and individual therapy. Majority of patients (54%) reported improvement following therapeutic intervention. Only 15% did not show clear improvement.

**Psychiatric input:** Psychiatry input was requested for 35% of cases for diagnostic assessment of Tourette’s syndrome and comorbid ADHD. Medications were considered in only 17% of cases following psychiatric assessment.

**Conclusions:**

The newly introduced Tic Disorder pathway successfully engaged patients, with high rates of therapy provision and evidence of clinical benefit. Only a minority of patients needed medication management for their tics.

**Disclosure of Interest:**

None Declared

